# Dionine: A New Ocular Analgesic

**Published:** 1904-06-04

**Authors:** 


### DIONINE: A NEW OCULAR ANALGESIC.

DIONINE is a white, odourless, crystalline powder, freely soluble in water. It may be used as a 5-per-cent. solution or ointment, the latter made with vaseline. Applied to the eye it does not modify in any way the sensitiveness of the conjunctiva or cornea, that is, it has no anæsthetic action. But in painful affections of the eyeball, such as iritis, iridocyclitis, glaucoma, ulcer of the cornea, etc., dionine solution or ointment will lessen or even entirely remove the pain, and the relief will persist for several hours. In respect both to its efficacy in relieving pain and the duration of its analgesic action, dionine is superior both to cocaine and holocaine. Like the latter, it does not raise the ocular tension, and therefore it is not, as is the case with cocaine, contra indicated in glaucoma. The only disadvantage attending its use is that sometimes after the first instillation there appears an intense chemosis. If the drug is persevered with, however, this soon subsides and does not recur after the later applications. In cases where the pain is slight and the inflammatory condition not very acute, it is sufficient to use a 2 per-cent. solution or ointment, and in this strength chemosis is never produced. The above results are recorded by Hinshelwood [1] as the result of observations made at the Glasgow Eye Infirmary.

[1] Brit. Med. Jour., April 30, 1904.

